# Clinical characteristics and prognosis of multiple myeloma with bone-related extramedullary disease at diagnosis

**DOI:** 10.1042/BSR20171697

**Published:** 2018-05-28

**Authors:** Chen Tian, Lu Wang, Ling Wu, Lei Zhu, Wengui Xu, Zhaoxiang Ye, Zhigang Zhao, Yafei Wang, Yizhuo Zhang

**Affiliations:** Tianjin Medical University Cancer Institute and Hospital, National Clinical Research Center for Cancer, Key Laboratory of Cancer Prevention and Therapy, Tianjin, China

**Keywords:** bone-related extramedullary disease, multiple myeloma, prognosis, PET-CT

## Abstract

Multiple myeloma (MM) is a hematological neoplasm which results in diffuse or focal bone infiltration and extramedullary lesions. It’s reported that infiltration of organs by plasma cells indicated worse prognosis, but the prognosis of patients with bone-related extramedullary disease (bEMD) is still unknown. One hundred and fourteen newly diagnosed MM patients were retrospectively reviewed. Results showed that the clinical features, overall survival (OS), and progression-free survival (PFS) of patients with and without bEMD had no statistical significance. Rib (46.1%) and vertebrae (17.9%) are common sites bEMD involved. Patients with diffuse bEMD had worse prognosis compared with patients with focal bEMD. Bisphosphonates played an important role in prolonging the survival of patients with bEMD. Positron emission tomography (PET)/computed tomography (CT) is sensitive in discovering bEMD than whole body low dose CT suggesting PET/CT to be a promising technique for initial staging. High β2-microglobulin and low albumin indicated shorter survival in patients with bEMD.

## Introduction

Multiple myeloma (MM) is a hematological neoplasm characterized by the clonal proliferation of malignant plasma cells in the bone marrow [1,2]. MM results in bone destruction and extramedullary lesions, which are defined as extramedullary disease (EMD). EMD is a type of MM defined by the presence of extraskeletal clonal plasma cell infiltration. EMD can be present at the time of either diagnosis (primary EMD) or relapse (secondary EMD) [3,4].

Clinically, three types of extramedullary lesions can be described: (a) tumor mass adjacent to bone and extending into soft tissue, (b) soft tissue or visceral tumor that is not connected to the bone, or (c) diffuse infiltration of organs by plasma cells without any obvious focal lesion [5,6]. However, the majority of studies don’t discriminate among the three types of EMD. With increasing awareness of this malignancy, some researchers adopted a strict definition of EMD, namely strict EMD (sEMD), with plasmacytomas deriving from hematogenous spread [7]. The last two types are sEMD. The first type is defined as bone-related EMD (bEMD).

It is reported that sEMD is frequently associated with poor outcome and resistance to treatment [8]; however, there’s no report on bEMD to the prognosis of MM. To illuminate the problem, we performed a comparison on clinical and laboratory features and prognosis between patients with and without bEMD to investigate whether patients suffering bEMD share the common clinical characteristics and outcome with patients without bEMD.

## Methods

### Patient samples

Patients diagnosed with MM were retrospectively reviewed at Tianjin Medical University Cancer Institute and Hospital from January 2000 to December 2015. Patients who lost follow-up or lack indispensable medical documents were excluded. All subjects gave informed consent, and the study protocol was approved by the Ethics Committee of Tianjin Medical University Cancer Institute and Hospital. All the patients had symptomatic myeloma requiring treatment. EM was diagnosed using imaging methods such as whole body low dose computed tomography (CT) or ^18^F-fluorodeoxyglucose (FDG) positron emission tomography (PET). One hundred and fourteen patients were finally enrolled, including 36 patients suffering bEMD at diagnosis and 78 patients without bEMD.

### Treatment and follow-up

All patients in the study received antimyeloma chemotherapy, including thalidomide (T) containing regimen, and bortezomib (B) containing regimen. Forty nine (43.0%) patients were treated with bortezomib containing regimen for first-line therapy, of which 10 (20.4%) patients with bEMD, 39 (79.6%) patients without bEMD. Sixty-five patients (57.0%) received thalidomide combined with chemotherapy such as adriamycin and cyclophosphamide, of which 26 (22.8%) patients with bEMD and 39 (34.2%) patients without bEMD.

Overall survival (OS) was calculated from the date of diagnosis until the date of death from any cause or until the date of final follow-up. Progression-free survival (PFS) was determined for responders from the time of diagnosis until relapse or death from any cause.

### Statistical analysis

Statistical analysis was performed using SPSS 17.0 (SPSS Inc., Chicago, IL, U.S.A.), and statistical significance was considered to be *P*<0.05. The two groups were compared by the *X*^2^ test and *T* test. The curves of OS and PFS were computed using the Kaplan–Meier method, and statistical differences between the curves were assessed by the log-rank test. Multivariate analysis was performed using the Cox proportional hazards regression model.

## Results

### Clinical characteristics of MM patients with and without bEMD

Of the 114 patients, 67 (58.8%) patients were male, and 47 (41.2%) patients were female. The median age of patients was 59 (range from 37 to 81 years old). The patients were divided into two groups: 78 patients without EMD, including both bEMD and sEMD, and 36 with bEMD. The clinical features of patients between the two groups are listed in Table 1 (*P*>0.05).

**Table 1:** Patients’ characteristics

Characteristics	Without EMD	With bEMD	*P* value
Gender M/F	44/34	23/13	*P*>0.05
Age ≥60/<60	42/36	19/17	*P*>0.05
IgG/IgA/IgD/light chain/nonsecretary	37/19/1/18/3	17/9/0/10/0	*P*>0.05
DS I/II/III	3/6/68	2/4/30	*P*>0.05
ISS I/II/III	10/29/33	8/10/14	*P*>0.05
Anemia (hemoglobin <100 g/l)	57	28	*P*>0.05
LDH high (>250 U/l)	11	7	*P*>0.05
Ca^2+^ high (>2.75 mmol/l)	13	6	*P*>0.05
β2-MG high (>2.7 mg/l)	55	29	*P*>0.05
Albumin low (<40 g/l)	33	12	*P*>0.05
Cytogenetics abnormal	22	6	*P*>0.05
Plasma cell in peripheral blood >60%	29	11	*P*>0.05

Abbreviations: β2-MG, β2-microglobulin; LDH, lactate dehydrogenase.

### Common sites of bEMD involvement

In the study, we found that rib was the most common sites involved in patients with bEMD, followed by vertebra (17.9%), skull (12.8%), sternum (10.3%), humerus (5.1%) and ilium (5.1%), which were listed in Table 2.

**Table 2:** Comparison of lesion detection rate in different bone areas

	Number	Ratio
Rib	18	46.1%
Vertebra	7	17.9%
Skull	5	12.8%
Sternum	4	10.3%
Humerus	2	5.1%
Ilium	2	5.1%
Pubis	1	2.6%

### Treatment strategies

All the patients received chemotherapy. Bortezomib-containing regimens, thalidomide-containing regimens, and conventional chemotherapy were given to 49 (43.0%, including 10 with bEMD and 39 without EMD), 51 (44.7%, including 20 with bEMD and 31 without EMD), and 14 (12.3%, including 6 with bEMD and 8 without EMD). Difference in the treatment regimens of bEMD group and no-EMD group showed no statistically significant (*P*=0.081).

A total of 11 (9.6%) patients received radiation therapy (XRT), of which 4 patients (36.4%) with bEMD, and 7 (had lytic destruction of the bone) without EMD (63.6%). Thirty patients (26.3%) received autologous hematopoietic stem cell transplantation (ASCT), of which 7 (23.3%) patients with bEMD and 23 without EMD (76.7%). One hundred and nine patients received bisphosphonates to inhibit bone destruction including 88 (32 with bEMD and 56 without EMD) patients using zoledronic acid and 21 patients (3 patients with bEMD and 18 patients without EMD) using pamidronate sodium. There was no statistically significant difference between the two groups of patients with and without bEMD (XRT: *P*=0.986; ASCT: *P*=0.258; bisphosphonate: *P*=0.052).

### Prognosis of MM patients with bEMD

**Figure 1:**
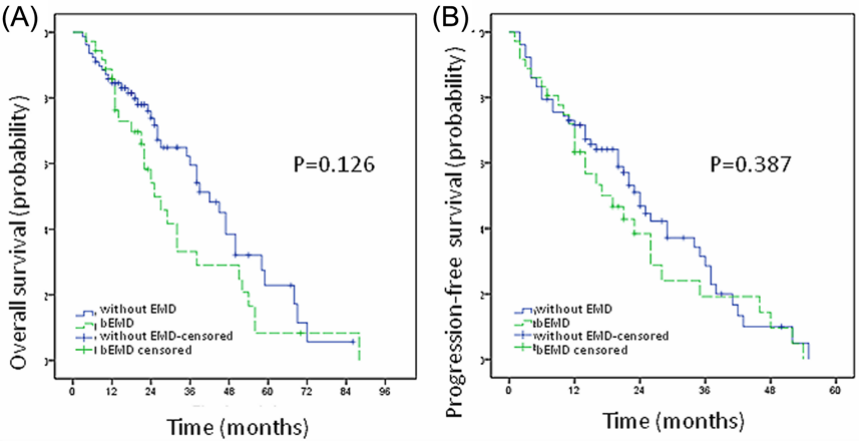
Comparison of prognosis of MM patients with and without bEMD The 5-year survival rate of patients with and without bEMD was 16.6% and 22.9% respectively, showing no significant difference (*P*=0.126, **A**). The 3-year PFS rate was 19.2% and 28.6% for patients with and without bEMD, showing no significant differences between the two groups (*P*=0.387, **B**).

The overall response rate of MM patients with and without bEMD was 80.6% and 85.9% respectively, showing no significant difference in curative effect between the two groups (*P*=0.581). Besides, there was no difference in complete response (CR) rate and partial response (PR) rate between these two groups (data not shown). At the end of follow-up, 64 patients (56.1%) died, including 26 patients with bEMD (40.6%), and 38 patients without EMD (59.4%). The median OS of patients with bEMD was 25 months (4–88 months), while that of patients without EMD was 42 months (3–86 months). The 5-year survival rate of patients with and without bEMD was 16.6% and 22.9% respectively, showing no significant difference (*P*=0.126, Figure 1A). The median PFS of patients with and without bEMD was 17 months (1–54 months) and 24 months (2–55 months). The 3-year PFS rate was 19.2% and 28.6% for patients with and without bEMD, showing no significant differences between the two groups (*P*=0.387, Figure 1B).

### Time interval from symptoms to treatment is related to prognosis

**Figure 2:**
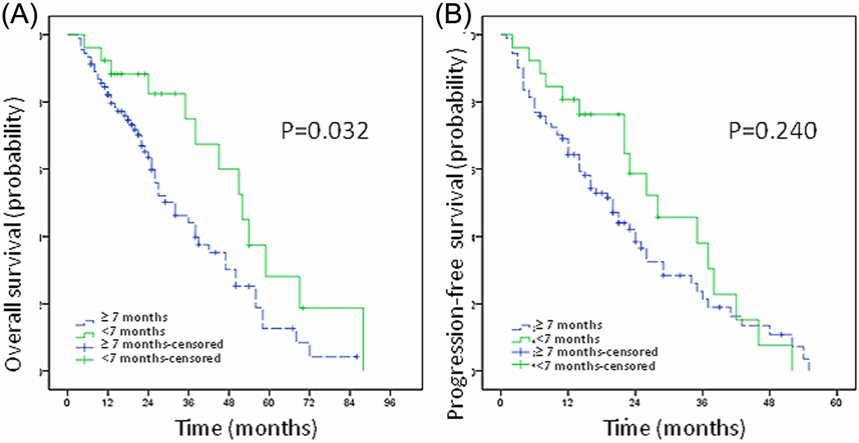
Time interval from symptoms to treatment is related to prognosis The OS of group A was significantly shorter than that of group B (*P*=0.032, **A**). The PFS between the two groups showed no significant difference (*P*=0.240, **B**).

The study showed that for all MM patients (with or without bEMD), the interval from clinical symptoms to the initiation of therapy was significantly related to prognosis. The patients were divided into two groups according to the time interval: group A: more than 7 months and group B: less than 7 months. Results showed that the median OS of group A was 32 months (3–86 months) and the 5-year OS rate was 12.6%. The median OS of group B was 52 months (5–88 months) and the 5-year OS rate 28.1%. The OS of group A was significantly shorter than that of group B (*P*=0.032, Figure 2A). The PFS between the two groups showed no significant difference (*P*=0.240, Figure 2B).

### Bisphosphonate and prognosis

**Figure 3:**
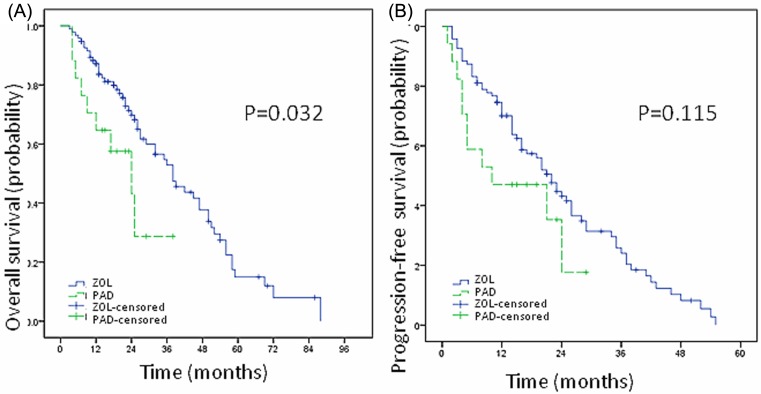
Zoledronic acid could inhibit bone destruction better than pamidronate disodium The median OS of ZOL group was 38 months (3–88 months), while that of PAD group was 24 months (4–38 months) respectively. There’s significant difference between these two groups suggesting that ZOL could inhibit bone destruction better than PAD (*P*=0.032, **A**). The median PFS of ZOL group was 22 months (2–55 months), while that of PAD group was 10 months (1–29 months) respectively, showing no significant difference (*P*=0.115, **B**).

Bisphosphonates play an important role in protecting bone integrity and preventing from bone-related adverse events in myeloma. In the present study, a total of 109 patients used bisphosphonates, including 88 (80.7%) patients using zoledronic acid (ZOL) and 21 (19.3%) patients using pamidronate disodium (PAD). The median OS and 3-year OS rate of ZOL group were 38 months (3–88 months) and 52.9%, while that of PAD group was 24 months (4–38 months) and 38.8% respectively. There’s significant difference between these two groups suggesting that ZOL could inhibit bone destruction better than PAD (*P*=0.032, Figure 3A). The median PFS and 3-year PFS rate of ZOL group was 22 months (2–55 months) and 24%, while that of PAD group was 10 months (1–29 months) and 17.6% respectively, showing no significant difference (*P*=0.115, Figure 3B).

### Increased detection sensitivity of bEMD by PET/CT compared with whole body low dose CT in newly diagnosed MM patients

The International Myeloma Working Group (IMWG) has revised the definition of MM with an aim of preventing end-organ damage by initiating systemic treatment before such damage occurs. PET/CT scans have emerged as an important modality that helps confirming diagnoses and leads to definitive therapy. We reviewed the imaging data of all patients to evaluate the difference of whole body low dose CT and PET/CT in the detection of bEMD. Of all patients receiving whole-body imaging assessment, 44 (48.9%) received whole-body low dose CT and 46 (51.1%) received PET/CT. The number of detectable bEMD is shown in Table 3. The bEMD detection rate by PET/CT was significantly higher than by whole body low dose CT in newly diagnosed MM patients (*P*=0.034).

**Table 3:** Comparison between PET/CT and whole body low dose CT

Detection	bEMD number
Whole body low dose CT	3.57 (0–15)
PET/CT	9.59 (1–25)

### Focal bEMD>3 predicted worse prognosis

**Figure 4:**
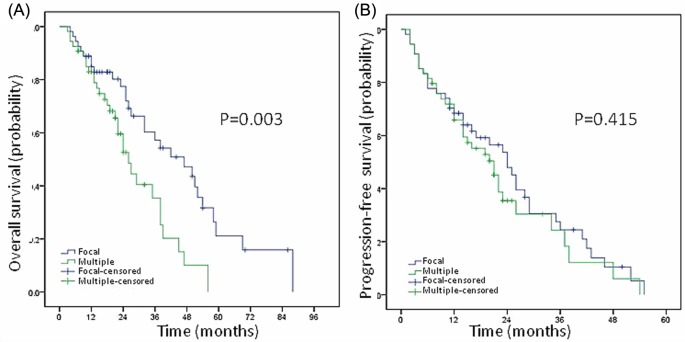
Multiple bEMD predicted worse prognosis The median OS of F group was 42 months while those of D group was 24 months, showing statistically significant difference between the two groups (*P*=0.003, **A**). The median PFS of F group and D group wa 24 and 20 months, showing no statistically significant difference between these two groups (*P*=0.415, **B**).

More than three focal bEMDs were defined as multiple bEMD. A total of 54 patients (50%) had multiple bEMD (named D group), and the remaining 54 (50%) patients had focal bEMD (named F group). The median OS and 5-year OS rate of F group were 42 months (4–88 months) and 21.1% while those of D group were 24 months (3–56 months) and 0%, showing statistically significant difference between the two groups (*P*=0.003, Figure 4A). The median PFS of F group and D group were 24 months (1–55 months) and 20 months (2–54 months). The 3-year PFS rate of F group and D group were 24.5% and 18.2%, showing no statistically significant difference between these two groups (*P*=0.415, Figure 4B).

### Low albumin expression was the independent prognostic factor for bEMD patients

In univariate analysis, we found that β2-microglobulin (β2-MG) > 2.7 mg/l (*P*=0.041) and albumin (ALB) < 35 g/l (*P*<0.001) were prognostic factors for MM patients with bEMD (Table 4). Importantly, multivariate Cox regression analysis showed that the level of ALB had a positive effect on OS and PFS (HR = 0.257, 95% CI: 1.445–4.572, *P*=0.001), indicating that ALB level is an independent risk factor for death.

**Table 4:** *P* values of all prognostic makers for overall survival as determined by univariate analysis

Factors	OS (*P* value)	PFS (*P* value)
ALB	<0.001	0.057
β2-MG	0.041	0.539

## Discussion

When extramedullary lesions of multiple myeloma were mentioned, most of the researchers defined that soft tissues are not connected to the bone and diffuse infiltration of organs as EMD [9–12]. EMD has been reported to predict a relatively shorter PFS and OS, both in newly diagnosed patients and in relapsed patients [13–16]. Although in the new drug era with bortezomib, EMD is still a huge problem in clinical treatment [17–20]. But what’s the clinical characteristics and prognosis of MM patients with extramedullary lesions adjacent to bone? There’s still no report.

The incidence rate of bEMD in MM was approximately 4.3–19.5% [21–23] while ribs, vertebrae, skull, sternum, humerus, ilium, and pubis are the common sites involved [24–27]. There were no significant differences in clinical characteristics, laboratory index, treatment response, and survival between patients with and without bEMD (*P*>0.05). Therefore, we believe that bEMD should be considered as a manifestation of tumor burden rather than a subtype of MM with different pathogenesis.

Bisphosphonates, particularly ZOL, can inhibit osteolysis and reduce skeletal tumor burden [28]. A large clinical trial showed that ZOL could reduce mortality (HR:0.84, 95% CI: 0.74–0.96; *P*=0.0118) and prolong the median OS for 5.5 months (*P*=0.04) compared with phosphonic acid [29]. Furthermore, ZOL can significantly improve the PFS (HR = 0.88, 95% CI: 0.80-0.98; *P*=0.0179) of patients [30]. Compared with PAD, the mortality risk of patients who received ZOL reduced 22% [31]. In the present study, we compared the OS and PFS of patients who received ZOL or PAD, showing significant difference in OS (*P*=0.032). The use of ZOL can prolong the OS of patients regardless of bEMD, suggesting it should be used as soon as possible.

PET/CT is proved to be a reliable technique for assessing skeletal involvement in multiple myeloma and a valuable tool at the onset of the disease for predicting outcomes in those patients who are eligible to subsequently receive autologous stem cell transplantation [32]. In the present study, the number of bEMD detected by whole body low dose CT was 3.75 (0–15), while the number of bEMD detected by PET/CT was 9.59 (1–25). PET/CT showed superior potential than CT (*P*=0.034) in detecting bone-related lesions. Our results found that patients with diffuse bone lesions had worse prognosis than those with focal lesions (*P*<0.05).

Previous reports found that age, DS stage, ISS stage, hemoglobin, platelet, plasma cell number in BM, β2-MG, albumin and lactate dehydrogenase (LDH) were prognostic factors for MM survival [33]. Multivariate Cox regression analysis showed that age, ISS stage and β2-MG were independent prognostic factors for MM survival [34–36]. But these studies did not distinguish patients with bEMD or not. The purpose of our study was to investigate the prognostic factors between patients with or without bEMD. Results showed that β2-MG and ALB were prognostic factors for survival in patients with bEMD and ALB level was an independent prognostic factor after Cox regression analysis.

## Conclusion

Although many researches focusing on the prognosis of MM, they do not distinguish whether the patients are accompanied by bEMD. Our study found that the clinical features, OS, and PFS of patients with and without bEMD had no statistical significance. Multiple bEMD predicted worse prognosis compared with focal bEMD. PET/CT is sensitive in discovering bEMD than whole body low dose CT suggesting PET/CT to be a promising technique for initial staging. High β2-MG and low ALB indicated shorter survival in patients with bEMD. Our cohort of patients is limited, and it is a retrospective study. It was the limitation that we couldn’t be sure whether our conclusion was applicable in other or larger cohorts.

## Abbreviations

β2-MG: β2-microglobulin
ALB: albumin
ASCT: autologous hematopoietic stem cell transplantation
bEMD: bone-related extramedullary disease
CT: computed tomography
EMD: extramedullary disease
LDH: lactate dehydrogenase
MM: multiple myeloma
OS: overall survival
PET: positron emission tomography
PFS: progression-free survival
sEMD: strict EMD
ZOL: zoledronic acid
